# Validation Study of the Petricore™ Aerobic Count (AC) for the Enumeration of Mesophilic Aerobic Bacteria in a Broad Range of Foods and Select Environmental Samples: AOAC *Performance Tested Method*^SM^ 032502

**DOI:** 10.1093/jaoacint/qsaf064

**Published:** 2025-06-28

**Authors:** Wonkee Sung, Young-Hee Cho, Sujeong Moon, Kateland Lanzit, M Joseph Benzinger, Benjamin Bastin, Erin Crowley

**Affiliations:** PNG BIOMED Co., Ltd, Unit 218, 120, Heungdoekjungang-ro, Giheung-gu, Yongin-si, Gyeonggi-do 16950, Republic of Korea; PNG BIOMED Co., Ltd, Unit 218, 120, Heungdoekjungang-ro, Giheung-gu, Yongin-si, Gyeonggi-do 16950, Republic of Korea; PNG BIOMED Co., Ltd, Unit 218, 120, Heungdoekjungang-ro, Giheung-gu, Yongin-si, Gyeonggi-do 16950, Republic of Korea; Q Laboratories, Inc, 1930 Radcliff Drive, Cincinnati, OH 45204, USA; Q Laboratories, Inc, 1930 Radcliff Drive, Cincinnati, OH 45204, USA; Q Laboratories, Inc, 1930 Radcliff Drive, Cincinnati, OH 45204, USA; Q Laboratories, Inc, 1930 Radcliff Drive, Cincinnati, OH 45204, USA

## Abstract

**Background:**

The Petricore™ Aerobic Count (AC) method is used for enumeration of mesophilic bacterial colony counts in a broad range of food and environmental samples. The plate is a ready-to-use dry rehydratable film media composed of modified standard-method nutrients, water-soluble gelling agents and a tetrazolium indicator on the adhesive sheets, and transparent cover film.

**Objective:**

The purpose of this study is to validate the Petricore AC for AOAC *Performance Tested Methods*^SM^ (PTM) certification.

**Methods:**

Matrix studies were conducted on a broad range of foods and select environmental samples: fresh raw ground beef, fresh raw ground pork, raw bacon, raw shrimp, raw salmon, frozen raw tuna, frozen sliced mushrooms, frozen avocado, frozen blueberries, bacon-lettuce-tomato sandwich, frozen pizza (margherita), cooked sausage (fish and chicken breast), Romaine lettuce, cabbage, fresh green juice, stainless steel surface, plastic surface, and lettuce wash water. Petricore AC results were compared to standard-method plating procedure results appropriate to each matrix type. Product consistency and stability testing was performed on three production lots of Petricore AC, and robustness experiments examined the allowable range of three parameters: culture temperature, incubation time, and inoculum amount.

**Results:**

In the matrix study, equivalent results were observed between the Petricore AC method and reference methods for all matrixes evaluated. The mean log_10_ differences between candidate method and reference methods were within the ranged from −0.23 to 0.35 log_10_ within the acceptable range of −0.50 to 0.50 log_10_. The range of standard deviation values of the candidate method (0.01–0.88 log_10_) and the reference method (0.02–0.91 log_10_) were similar in all matrixes evaluated. The range of correlation factor *R*^2^ was between 0.9539 and 0.9981, indicating strong correlation between the two methods. In the product consistency/stability study, the Petricore AC plate was proven to be equivalent across production lots, and the shelf life was established at 1 year. Small differences in method parameters did not affect the Petricore AC results in robustness testing.

**Conclusion:**

The Petricore AC plate is an accurate method for the enumeration of mesophilic aerobic bacteria in the matrixes evaluated.

**Highlights:**

The data were reviewed by the AOAC PTM Program and approval was granted for certification of Petricore AC as PTM 032502.

Aerobic microbial count is used to assess microbial contamination in food and food manufacturing facilities and can be used as a sanitary hygiene and process indicator in Hazard Analysis and Critical Control Point programs (1, 2). Aerobic bacteria are routinely tested as an assessment of the general microbiological quality of foods and as a potential shelf-life predictor. Any surfaces used for food production may also be assessed for a level of aerobic bacteria to provide a general indication of the cleanliness of food production areas and of equipment. The Petricore Aerobic Count (AC) is a ready-to-use, pre-made alternative to standard-method plating media, designed to enumerate total aerobic bacteria from foods and environmental samples.

## Principle 

Petricore AC is a ready-to-use dry rehydratable film media with a transparent cover, which is composed of modified standard-methods nutrients, water-soluble gelling agents, and a tetrazolium indicator. Petricore AC is intended to determine the total aerobic colony counts in the food and beverage industries. Petricore AC simplifies the workflow and reduces the amount of waste disposal.

## Scope of Method

- *Target organism*.*—*Mesophilic aerobic bacteria.
- *Matrixes*.*—*Fresh raw ground beef (50 g), fresh raw ground pork (50 g), raw bacon (50 g), raw shrimp (50 g), raw salmon (50 g), frozen raw tuna (50 g), frozen sliced mushrooms (50 g), frozen avocado (50 g), frozen blueberries (50 g), bacon-lettuce-tomato sandwich (50 g), frozen pizza (margherita; 50 g), cooked sausage (fish and chicken breast; 50 g), Romaine lettuce (50 g), cabbage (50 g), fresh green juice (50 mL), stainless steel surface (4” × 4”, sponge), plastic surface (4” × 4”, sponge), and lettuce wash water (50 mL).
- *Summary of validated performance claims*.*—*The study data indicate with 95% confidence that the Petricore AC method is equivalent to the U.S. Department of Agriculture (USDA) Food Safety and Inspection Service *Microbiology Laboratory Guidebook* (MLG) 3.02 Quantitative Analysis of Bacteria in Foods as Sanitary Indicators (3) for fresh raw ground beef, fresh raw ground pork, raw bacon, and cooked sausage, to the U.S. Food and Drug Administration (FDA) *Bacteriological Analytical Manual* (BAM) Chapter 3, Aerobic Plate Count (4) for raw shrimp, raw salmon, frozen raw tuna, frozen sliced mushrooms, frozen avocado, frozen blueberries, bacon-lettuce-tomato sandwich, frozen pizza, Romaine lettuce, cabbage, fresh green juice, stainless steel surface, and plastic surface, and to ISO 4833-1:2013/2022, Microbiology of food and animal feeding stuffs—Horizontal methods for the enumeration of microorganisms—Part 1: Colony count at 30°C by the pour plate technique with Amendment 1: Clarification of Scope (5) for lettuce process water.

## Materials and Methods

### Test Kit Information

- *Kit name*.*—*Petricore AC
- *Catalog number*.*—*2500
- *Ordering information*.—PNG BIOMED Co., Ltd, Unit 218, 120, Heungdoekjungang-ro, Giheung-gu, Yongin-si, Gyeonggi-do, 16950, Republic of Korea. E-mail: strategy@pngbiomed.com; tel: +82-31-627-2331; fax: +82-31-627-2330; website: www.pngbiomed.com.

### Test Kit Components

*Petricore AC plate*.*—*One package containing 50 ready-to-use film plates.

### Additional Supplies and Reagents

- *Butterfield's phosphate diluent.—*Cat. No. 053014 (HAPS) or equivalent.
- *Dey/Engley* (*D/E) neutralizing broth—*Cat. No. SSL10DE (Neogen) or equivalent.
- *Filter laboratory blender bags*.*—*Cat. No. B01318, B01416, or B01525 (Nasco) or equivalent.
- *Serological pipettes*.—Aerosol resistant for sampling and delivering 1 mL.
- *Plate spreader*.*—*Cat. No. 6496 (Neogen) or equivalent.

### Apparatus

- *Incubator*.*—*Jeiotech, capable of maintaining 35 ± 1°C, or equivalent.
- *Blender.—*Waring 8011, high-speed (10 000–12 000 rpm), minimum capacity 0.5 L, or equivalent.
- *Laboratory paddle blender*.*—*Seward 400, 3500 or equivalent, for sample homogenization, or equivalent.
- *Vortex mixer.—*IKA 3340000 or equivalent.

### Safety Precautions

The Petricore AC method components are not hazardous. However, if medium or reagent comes into contact with eyes or mouth, immediately wash with water and consult a physician. Laboratory personnel should follow appropriate laboratory safety precautions and have ready access to associated material safety data sheets. After use, the plates may contain microorganisms that may be a potential biohazard. All waste must be sterilized and disposed of in a biohazard bag. Follow the local industry standards for disposal.

### Sample Preparation

- *For solid food products*.*—*Weigh 50 g test portion aseptically. Homogenize the test portion with 450 mL Butterfield's Phosphate-Buffered Diluent (BPBD) thoroughly. If needed, prepare 10-fold serial dilutions in BPBD to achieve the counting range on the Petricore AC plate.
- *For liquid products*.*—*Weigh 50 mL test portion aseptically. Mix the test portion with 450 mL BPBD thoroughly. If needed, prepare 10-fold serial dilutions in BPBD to achieve the counting range on the Petricore AC plate.
- *For environmental surfaces (4” × 4”, sponges)*.*—*Use a sponge which has been pre-hydrated with 10 mL D/E neutralizing broth and sample the surface by sponging back and forth 10 times horizontally and 10 times vertically. Add 90 mL Butterfield’s phosphate diluent to sponges and homogenize by hand carefully for 30–60 s. If needed, prepare 10-fold serial dilutions in BPBD to achieve the counting range on the Petricore AC plate.
- *For optimal growth of microorganisms*.—Adjust the pH of the sample suspension to neutral pH (pH 7.0 ± 0.4). Adjust the pH with 1 N NaOH for acidic products and 1 N HCl for alkaline products.

### Analysis

- Place Petricore AC plate on a flat surface.
- Gently remove the cover film without touching the media.
- Align the pipette perpendicularly. Inoculate 1 mL of the appropriate dilution onto the center of the bottom film.
- Replace the cover film.
- Place the spreader on the inoculum of the Petricore AC plate and press gently to distribute the inoculum evenly.
- Leave the plates on the flat surface for at least 30 s for gel formation.
- Stack the plates no more than 20. Incubate plates at 35 ± 1°C for 48 ± 2 h.

### Interpretation

- Count the total red colonies on the plate. The countable range for Petricore AC is 15–300 colony-forming units (CFU)/plate. Calculate CFU by multiplying the total count by the appropriate dilution factor.
- If colonies exceed 300, count the average number of colonies in one square (1 cm^2^) and multiply it by 20 to determine the estimated count.
- The entire growth area can appear red without colonies if a very high concentration of bacteria is present on the plates. Record the results as “too numerous to count (TNTC).”

### Experimental

The validation study was conducted under the AOAC Research Institute *Performance Tested Methods*^SM^ program following the *AOAC INTERNATIONAL Methods Committee Guidelines for Validation of Microbiological Methods for Food and Environmental Surfaces* (6). Method developer studies were conducted in the laboratories of PNG BIOMED Co., Ltd (Yongin-si, Korea) and included the matrix study for all claimed matrixes, product consistency and stability study, and robustness testing. The independent laboratory study was conducted by Q Laboratories (Cincinnati, OH, USA) and included a matrix study for fresh raw ground beef (approximately 80% lean), raw frozen tuna, Romaine lettuce, and stainless steel surface (4” × 4’, sponges).

**Table 1.** Categories, items, and reference methods for the matrix study

Category^a^	Items	Reference method
Raw and ready-to-cook meat	Raw ground beef, raw ground pork, raw bacon	MLG 3.02
Raw and ready-to-cook fish and seafood	Raw shrimp, raw salmon, frozen raw tuna	BAM Ch. 3
Processed fruits and vegetables	Frozen sliced mushrooms, frozen avocado, frozen blueberries	BAM Ch. 3
Multicomponent foods or meal components	Bacon-lettuce-tomato sandwich, frozen pizza (margherita), cooked sausage (fish and chicken breast)	BAM Ch. 3 (BLT and pizza), MLG 3.02 (sausage)
Fresh produce and juice	Romaine lettuce, cabbage, fresh green juice	BAM Ch. 3
Environmental samples (food or feed production)	Stainless steel surface, plastic surface, lettuce wash water	BAM Ch. 3 (surfaces) ISO 4833-1 (process water)

a Categories are based on ISO 16140–2 Annex A (7).

### Method Developer Studies

- *Matrix study*.—The Petricore AC method was evaluated for the enumeration of mesophilic aerobic bacteria for a broad range of foods and select environmental samples. Matrix categories are taken from Annex A in the ISO 16140-2:2016, Microbiology of the food chain—Method validation—Part 2: Protocol for the validation of alternative (proprietary) methods against a reference method (7). The matrix categories, food items, and reference methods are listed in Table 1. Fifteen foods from five categories and three environmental samples were tested and include fresh raw ground pork (shoulder), fresh raw ground beef, raw bacon (31% fat, 36% salt), thawed whole shrimp, raw salmon fillet (wild-caught), frozen raw tuna (yellowfin belly loin), frozen sliced mushrooms (white button mushroom), frozen avocado (diced and peeled), frozen blueberries, bacon-lettuce-tomato sandwich (28% fat, 21% salt), frozen pizza (margherita, 24% mozzarella cheese, 74% fat), cooked sausage (fish and chicken breast, 1% fat), romaine lettuce, cabbage (organic white cabbage), fresh green juice (apple 61.8%, white grape, kale, spinach 6.01%), stainless steel surface (tray), plastic surface (cutting board), and process water (lettuce wash water).
  All food matrixes were purchased from local suppliers. Each matrix was screened for the total aerobic plate count using the appropriate reference methods: MLG 3.02 for fresh raw ground pork, fresh raw ground beef, raw bacon, and cooked sausage; BAM Ch. 3 for raw shrimp, raw salmon, frozen raw tuna, frozen sliced mushrooms, frozen avocado, frozen blueberries, bacon-lettuce-tomato sandwich, frozen pizza, cooked sausage, Romaine lettuce, cabbage, fresh green juice, stainless steel surface, and plastic surface; and ISO 4833–1:2013/2022 for lettuce wash water.
  The food matrixes and lettuce wash water were found to be naturally contaminated with aerobic bacteria except for frozen avocado, frozen blueberries, and cooked sausage. These three matrixes required artificial contamination, as did the stainless steel and plastic environmental surfaces. Bacterial strains obtained from the American Type Culture Collection (ATCC, Manassas, VA, USA) were used for the inoculation. Frozen avocado was inoculated with *Staphylococcus aureus* (ATCC 25923), frozen blueberries were inoculated with *Escherichia coli* (ATCC 25922), cooked sausage was inoculated with *Salmonella* Typhimurium (ATCC 12028), stainless steel was inoculated with *Listeria monocytogenes* (ATCC 7644), and plastic was inoculated with *Salmonella Typhimurium* (ATCC 12028).
  For frozen avocado, blueberries, and cooked sausage, the bacterial strains were grown in Tryptic Soy Agar (TSB) broth and incubated for 24 ± 2 h at 35 ± 1°C. Each matrix chopped into small pieces and then split into four materials and chopped well. The strains were diluted in BPBD and added to the materials to target low (100–1000 CFU/g), medium (1000–10 000 CFU/g,) and high (10 000–100 000 CFU/g) contamination levels. One material was not inoculated to serve as the control. After inoculation, each material was mixed thoroughly using sterile utensils and then separated into five 50 g test portions per level. Each test portion was placed into a sterile sample bag. The avocado and blueberry test portions were placed at −20°C for 2 weeks prior to analysis. The cooked sausage was placed at 2–8°C for 48–72 h prior to analysis.
  For the stainless steel and plastic surfaces, each surface area was sterilized using an autoclave at 121°C for 15 min. For the stainless steel, the *L. monocytogenes* strain was diluted in brain heart infusion (BHI) broth to target a 100–1000 CFU/test area, 1000–10 000 CFU/test area, and 10 000–100 000 CFU/test area. A 1 mL aliquot of each level was applied to five 4” × 4” test areas. A 1 mL aliquot of diluent was applied to five 4” × 4” test areas to obtain a <10 CFU/test area level. The test areas were allowed to air-dry for 16–24 h prior to sampling.
  For matrixes that were naturally contaminated, samples from different brands and product lots were used to achieve three different microbial contamination levels. For low-level contamination, materials were stored under standard conditions, with refrigerated items maintained at 4–8°C and frozen items at −20°C. Medium-level contamination was achieved by mixing appropriate amounts of high- and low-level contamination materials. For high-level contamination, materials were temperature abused at 36°C for 15–18 h to reach a sufficient concentration.
  Each matrix was evaluated in a paired study design, comparing the Petricore AC with the appropriate reference method. For the food matrix, each 50 g or mL test portion was combined with 450 mL BPBD and homogenized by a paddle blender for matrixes compared to MLG 3.02, or by a blender for matrixes compared to BAM Ch. 3 for 2 min. The homogenate was further diluted in BPBD by adding 10 mL of the homogenate into 90 mL BPBD dilution blank. Further dilutions were carried out to ensure the results would be in the countable range. For lettuce wash water, each 50 mL test portion was combined with 450 mL BPBD and swirled until mixed well. Further dilutions were made in BPBD to obtain results in the countable range.
  For the surface test areas, each 4” × 4” surface area was sampled according to ISO 18593:2018, Microbiology of food and animal feeding stuffs—Horizontal methods for surface sampling (8). A sampling sponge was pre-moistened in 10 mL of D/E neutralizing broth. Each test area was sampled by using firm and even pressure 10 times diagonally, vertically, and horizontally. After sampling, the sponge was added to a sample bag, and 90 mL BPBD was added to the sponge and massaged for 1–2 min. Further dilutions were made in BPBD to obtain results in the countable range.
  For the paired comparison to MLG 3.02, dilutions from each contamination level for fresh raw ground pork, fresh raw ground beef, raw bacon, and cooked sausage were randomized and blind-coded. From each dilution, 1 mL was plated onto Petricore AC, and 1 mL was plated into each of two corresponding sterile Petri dishes. Molten plate count agar (PCA) was cooled to 45 ± 1°C in a water bath, and 12–15 mL was poured into the Petri dishes. The contents were swirled gently and allowed to harden before placing in the incubator. Petricore AC and PCA plates were incubated at 35 ± 1°C for 48 ± 2 h.
  For the paired comparison to BAM Ch. 3, dilutions from each contamination level for raw shrimp, raw salmon, frozen raw tuna, frozen sliced mushrooms, frozen avocado, frozen blueberries, bacon-lettuce-tomato sandwich, frozen pizza, Romaine lettuce, cabbage, fresh green juice, stainless steel surface, and plastic surface were randomized and blind-coded. From each dilution, 1 mL was plated onto Petricore AC, and 1 mL was plated into each of two corresponding sterile Petri dishes. Molten PCA was cooled to 45 ± 1°C in a water bath, and 12–15 mL was poured into the Petri dishes. The contents were swirled gently and allowed to harden before placing in the incubator. Petricore and PCA plates were incubated at 35 ± 1°C for 48 ± 2 h.
  For the paired comparison to ISO 4833-1:2013/2022, dilutions from each contamination level for lettuce wash water were randomized and blind-coded. From each dilution, 1 mL was plated onto Petricore AC, and 1 mL was plated into each of two corresponding sterile Petri dishes. Molten PCA was cooled to 45 ± 1°C in a water bath, and 12–15 mL was poured into the Petri dishes. The contents were swirled gently and allowed to harden before placing in the incubator. Petricore and PCA plates were incubated at 35 ± 1°C for 48 ± 2 h.
  Following incubation, plates were decoded and counted, and the duplicate PCA plates for the reference methods were averaged. The results were log_10_ transformed using the equation Log_10_[CFU/g + (0.1)f], where f is the reported CFU/unit corresponding to the smallest reportable result (in this case, 1). The mean and s_r_  were calculated for each concentration of each matrix. Results were analyzed for statistical difference between the Petricore AC results and the reference method results using difference of means (DOM) with 90% confidence interval (CI) according to the AOAC Least Cost Formulations (LCF) Quantitative analysis for micro methods v1.2 (Virginia Beach, VA, USA).
- *Product consistency/stability study.—*Three unique production lots were produced and used for stability testing and lot-to-lot variability testing. One lot was near the expiration date (1 year), one lot was near the middle of the expiration period (6 months), and one lot was newly manufactured (2 weeks). The artificially contaminated cooked sausage low and high levels, plus the non-inoculated level (five test portions each) from the matrix study were used for the study. Dilutions from each contamination level were randomized and blind-coded, and then 1 mL was plated onto the corresponding Petricore AC plate for each test lot. The plates were incubated at 35 ± 1°C for 48 ± 2 h. Following incubation, plates were decoded and counted, and the results were log_10_ transformed using the equation Log_10_[CFU/g + (0.1)f]. The mean and s_r_ were calculated for each lot at each concentration. Results were analyzed for statistical difference between lots using DOM with 90% CIs according to the AOAC LCF Quantitative analysis for micro methods v1.2.
- *Robustness study.—*Modest variations to method operating parameters were evaluated in the robustness study. Volume of sample homogenate delivered to the plate (0.95 and 1.05 mL), incubation time (44 and 52 h) and incubation temperature (33 and 37°C) tested. The parameters were combined into test combinations in a factorial design, and each test combination was compared to the nominal test condition, 1 mL sample homogenate, 48 ± 2 h, and 35 ± 1°C. The artificially contaminated cooked sausage low and high levels, plus the non-inoculated level (five test portions each) from the matrix study were used for the study. Dilutions from each contamination level were randomized and blind-coded, and then Petricore AC plates were inoculated and incubated according to the factorial design. Following incubation, plates were decoded and counted, and the results were log_10_ transformed using the equation Log_10_[CFU/g + (0.1)f]. The mean and s_r_ were calculated for each test combination and the nominal test condition. Results were analyzed for statistical difference between test conditions using DOM with 90% CIs according to the AOAC LCF Quantitative analysis for micro methods v1.2.

### Independent Laboratory Study

- *Matrix study*.—The independent laboratory evaluated fresh raw ground beef, raw frozen tuna, Romaine lettuce, and stainless steel surface. All food matrixes were purchased from a local supplier. The total aerobic plate count (APC) was pre-screened and determined following MLG 3.02 for raw ground beef and BAM Ch. 3 for raw frozen tuna and Romaine lettuce. An APC of 3.0 × 10^3^ CFU/g was obtained from the fresh raw ground beef, 3.3 × 10^3^ CFU/g from the frozen raw tuna, and 4.3 × 10^2^ CFU/g from the Romaine lettuce. The target levels of contamination evaluated consisted of low (100–1000 CFU/g), medium (1000–10 000 CFU/g,) and high (10 000–100 000 CFU/g). Each food matrix was further temperature abused to cover a 2–3 log range of contamination by storing at 25 ± 1°C for up to 5 h. The temperature-abused matrixes were then diluted with less contaminated matrixes and mixed thoroughly with a sterile spatula. For each contamination level, 50 g test portions were then weighed directly into sterile blender jars or filter laboratory blender bags.
  The stainless steel was supplied by the independent laboratory. The surface of the stainless steel was chemically disinfected by 70% ethanol solution prior to artificial contamination. The ethanol solution was allowed to sit for 10 min prior to being removed from the surface. The surface of the stainless steel was then artificially contaminated with *L. monocytogenes* (ATCC 7644). The inoculum was prepared by propagating a single *L. monocytogenes* strain from a stock culture stored at −70°C in a cryoprotectant (sterile glycerol), on Sheep blood agar (SBA) for 24 ± 2 h at 35 ± 1°C. A single colony from the SBA plate was then transferred into BHI broth and incubated at 35 ± 1°C for 24 ± 2 h. Following incubation, the *L. monocytogenes* strain was diluted to the following target levels using BPBD: 100–1000 CFU/test area, 1000–10 000 CFU/test area, and 10 000–100 000 CFU/test area. A 1 mL aliquot of each level was applied to 4” × 4” sample areas. A 1 mL aliquot of BPBD was applied to a 4” × 4” sample area to obtain a <10 CFU/test area level. The surface sample areas were allowed to air-dry for 16–24 h prior to sampling. Once dry, the surface was sampled with a pre-moistened sponge (10 mL of D/E neutralizing broth).
  For the paired comparison to MLG 3.02, dilutions from each contamination level for fresh raw ground beef were randomized and blind-coded. From each dilution, 1 mL was plated onto Petricore AC, and 1 mL was plated into each of two corresponding sterile Petri dishes. Molten PCA was cooled to 45 ± 1°C in a water bath, and 12–15 mL was poured into the Petri dishes. The contents were swirled gently and allowed to harden before placing in the incubator. Petricore and PCA plates were incubated at 35 ± 1°C for 48 ± 2 h. For the paired comparison to BAM Ch. 3, dilutions from each contamination level for frozen raw tuna, Romaine lettuce, and stainless steel surface were randomized and blind-coded. From each dilution, 1 mL was plated onto Petricore AC, and 1 mL was plated into each of two corresponding sterile Petri dishes. Molten PCA was cooled to 45 ± 1°C in a water bath, and 12–15 mL was poured into the Petri dishes. The contents were swirled gently and allowed to harden before placing in the incubator. Petricore and PCA plates were incubated at 35 ± 1°C for 48 ± 2 h.
  Following incubation, plates were decoded and counted, and the duplicate PCA plates for the reference methods were averaged. The results were log_10_ transformed using the equation Log_10_[CFU/g + (0.1)f]. The mean and s_r_ were calculated for each concentration of each matrix. Results were analyzed for statistical difference between the Petricore AC results and the reference method results using DOM with 90% CI according to the AOAC LCF Quantitative analysis for micro methods v1.2.

## Results and Discussion

**Figure 1.**
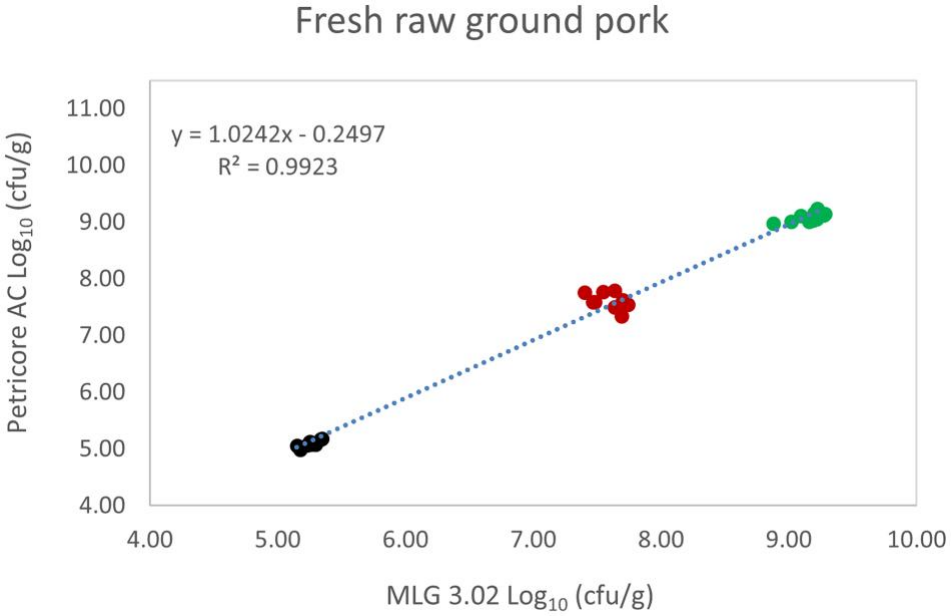
Petricore AC vs. MLG 3.02, fresh raw ground pork.

**Figure 2.**
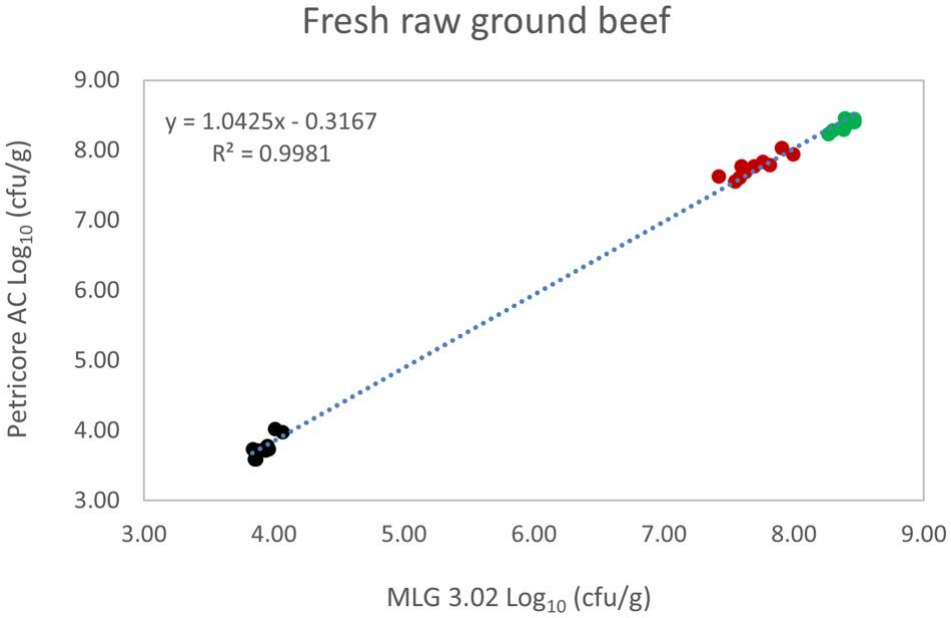
Petricore AC vs. MLG 3.02, fresh raw ground beef.

**Figure 3.**
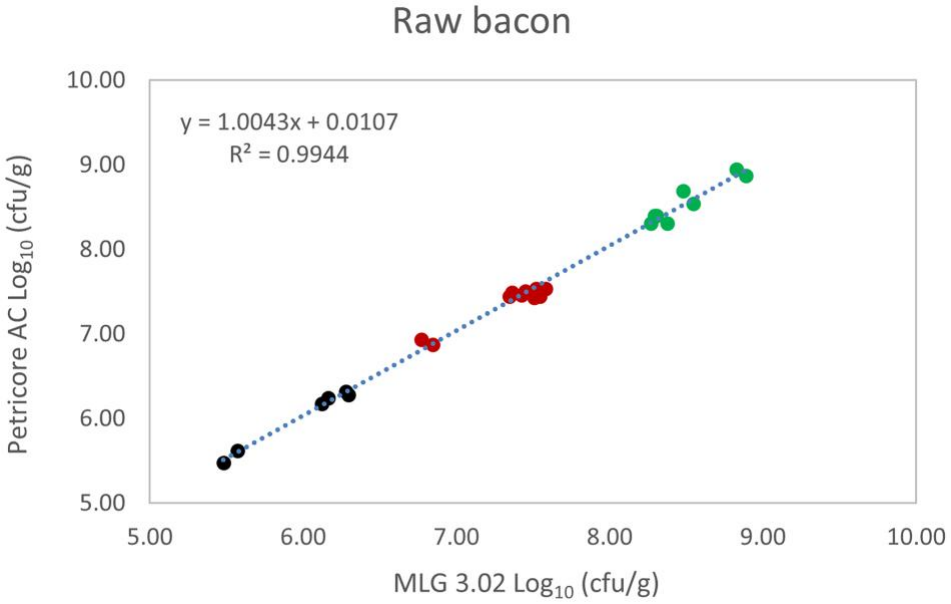
Petricore AC vs. MLG 3.02, raw bacon.

**Figure 4.**
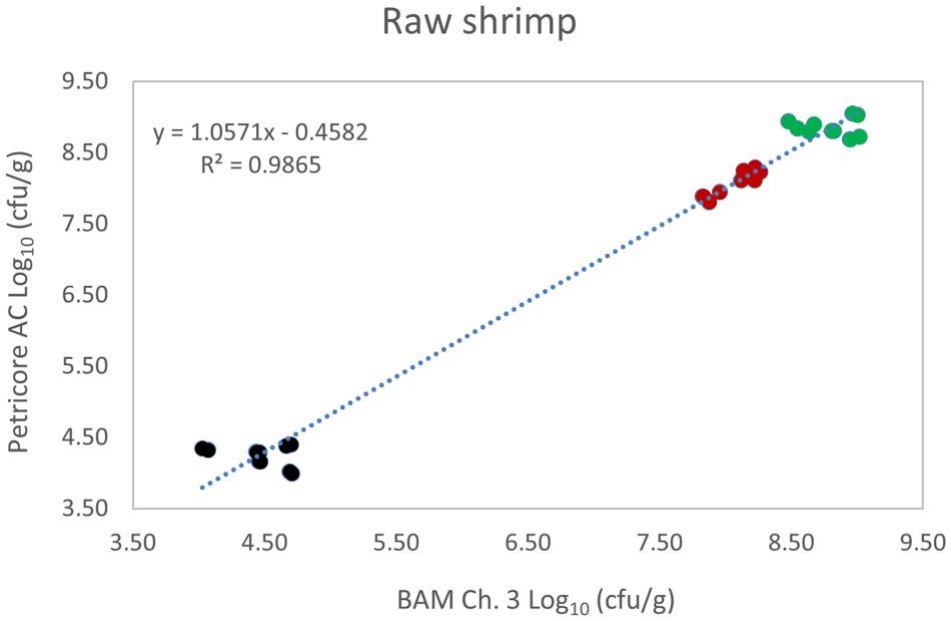
Petricore AC vs. BAM Ch. 3, raw shrimp.

**Figure 5.**
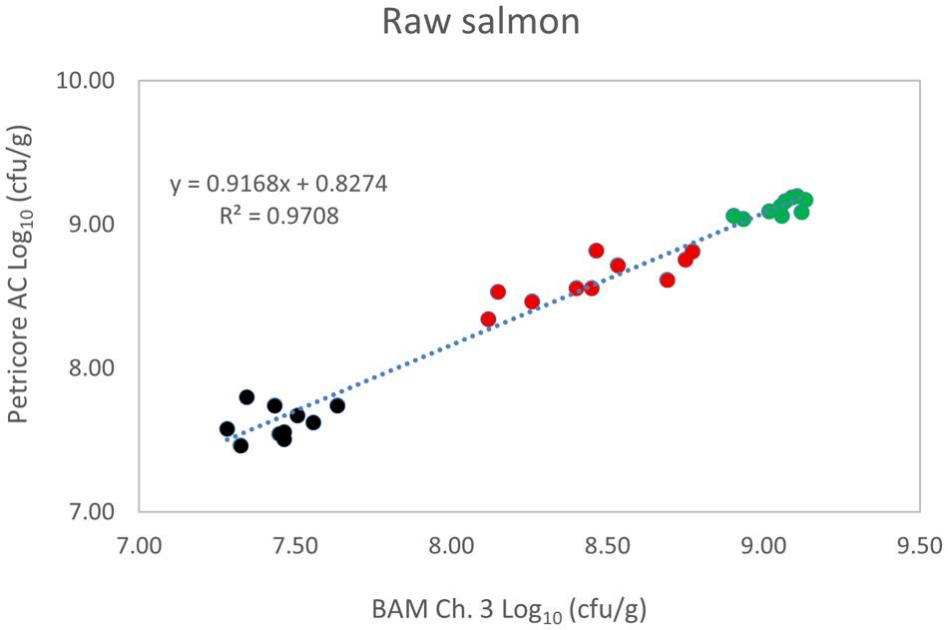
Petricore AC vs. BAM Ch. 3, raw salmon.

**Figure 6.**
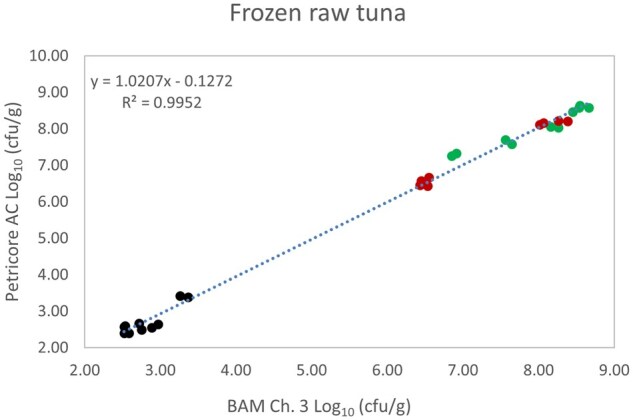
Petricore AC vs. BAM Ch. 3, frozen raw tuna.

**Figure 7.**
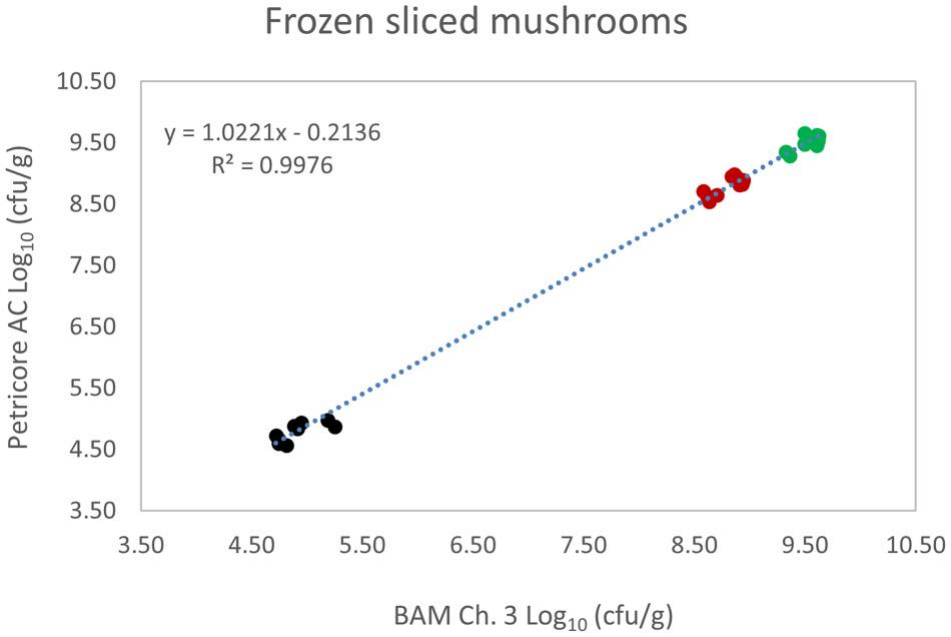
Petricore AC vs. BAM Ch. 3, frozen sliced mushrooms.

**Figure 8.**
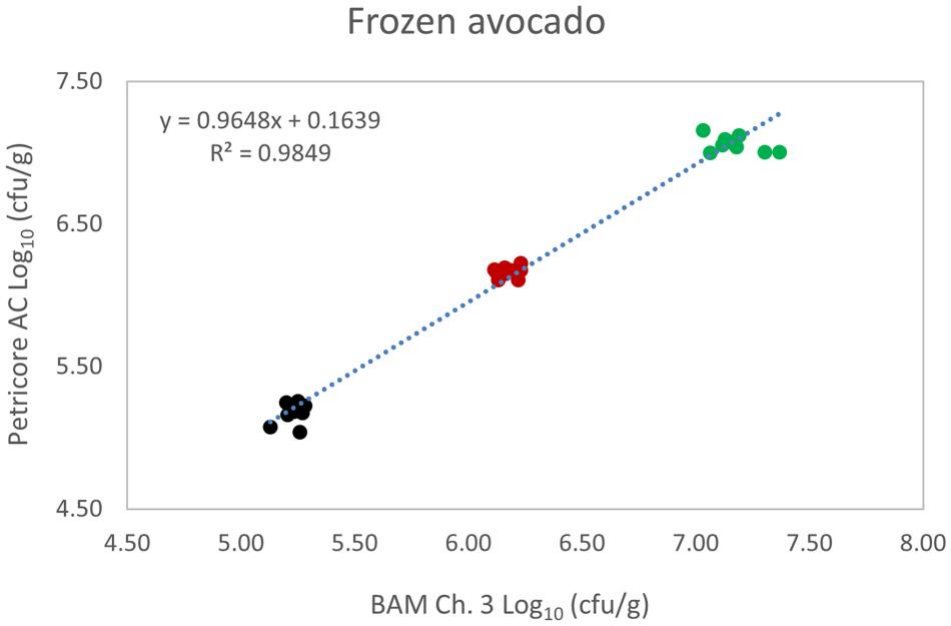
Petricore AC vs. BAM Ch. 3, frozen avocado.

**Figure 9.**
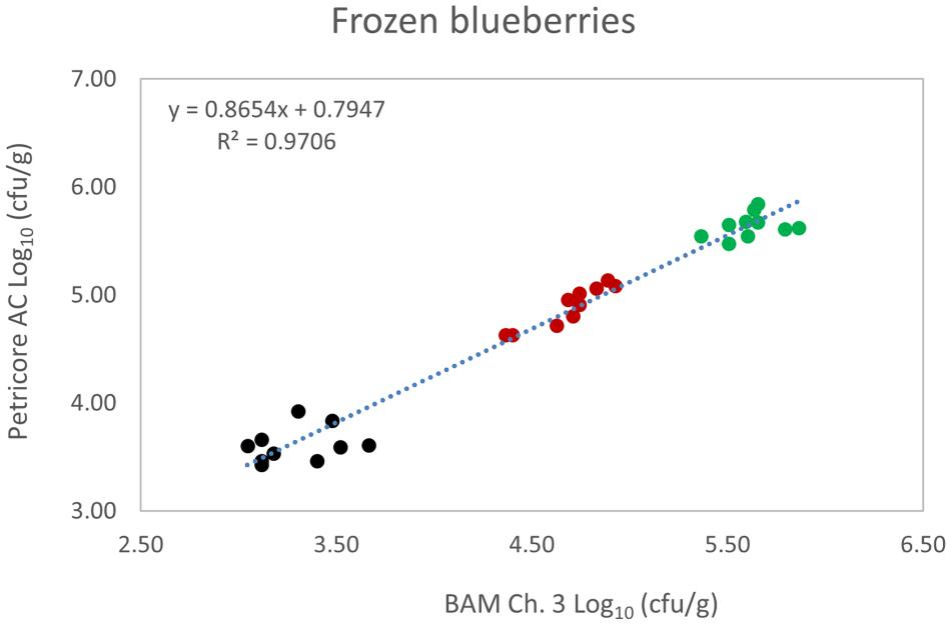
Petricore AC vs. BAM Ch. 3, frozen blueberries.

**Figure 10.**
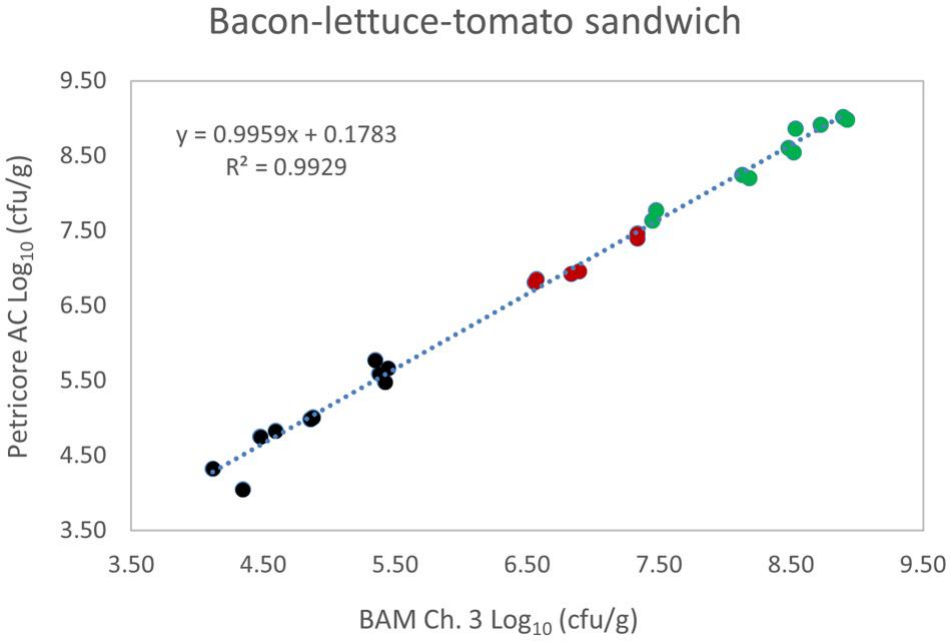
Petricore AC vs. BAM Ch. 3, bacon-lettuce-tomato sandwich.

**Figure 11.**
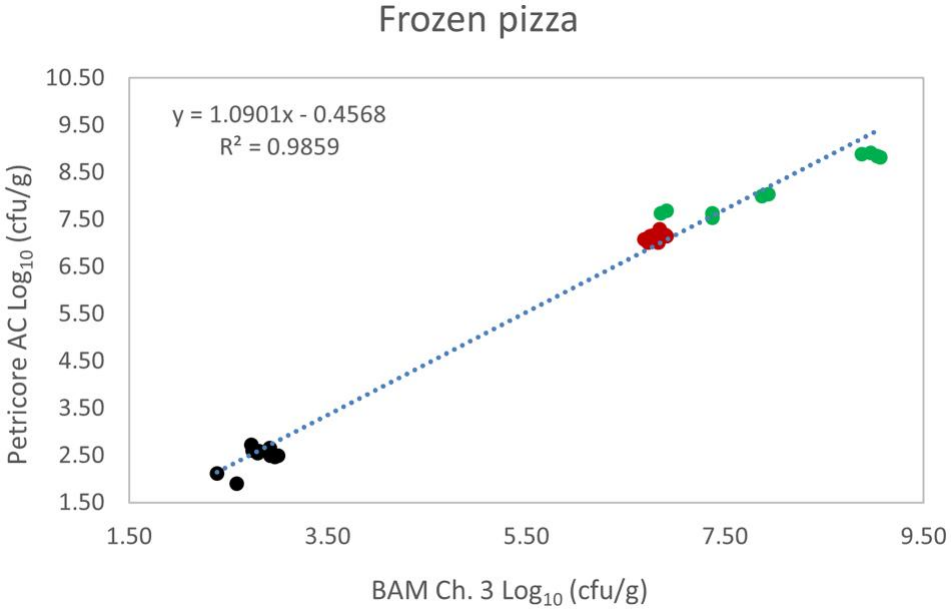
Petricore AC vs. BAM Ch. 3, frozen pizza.

**Figure 12.**
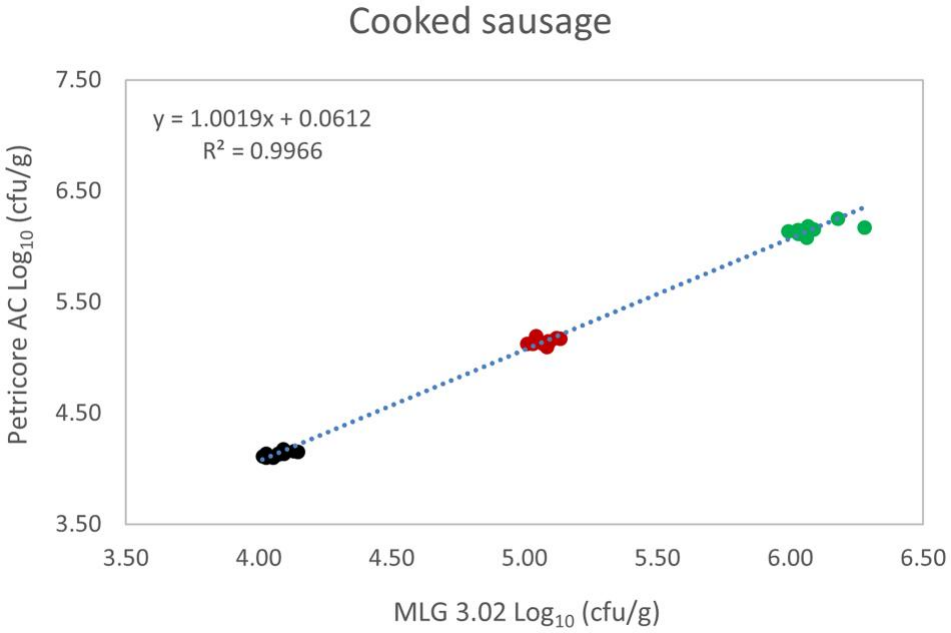
Petricore AC vs. MLG 3.02, cooked sausage.

**Figure 13.**
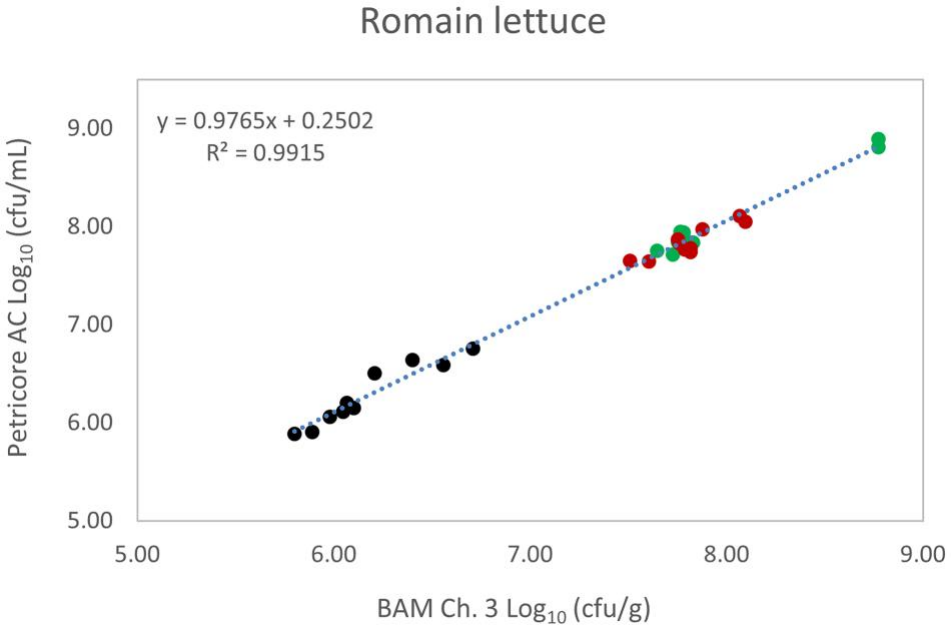
Petricore AC vs. BAM Ch. 3, Romaine lettuce.

**Figure 14.**
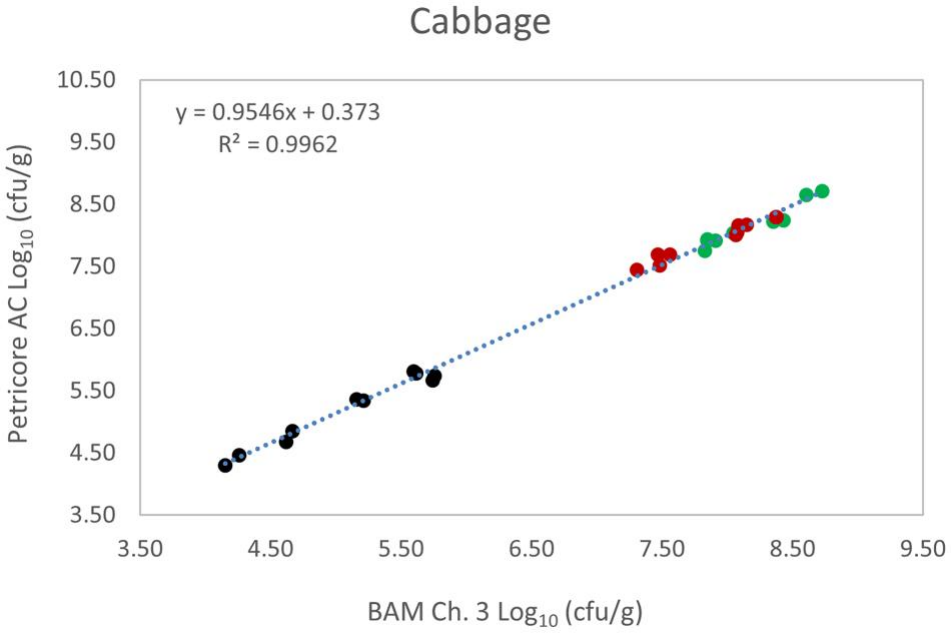
Petricore AC vs. BAM Ch. 3, cabbage.

**Figure 15.**
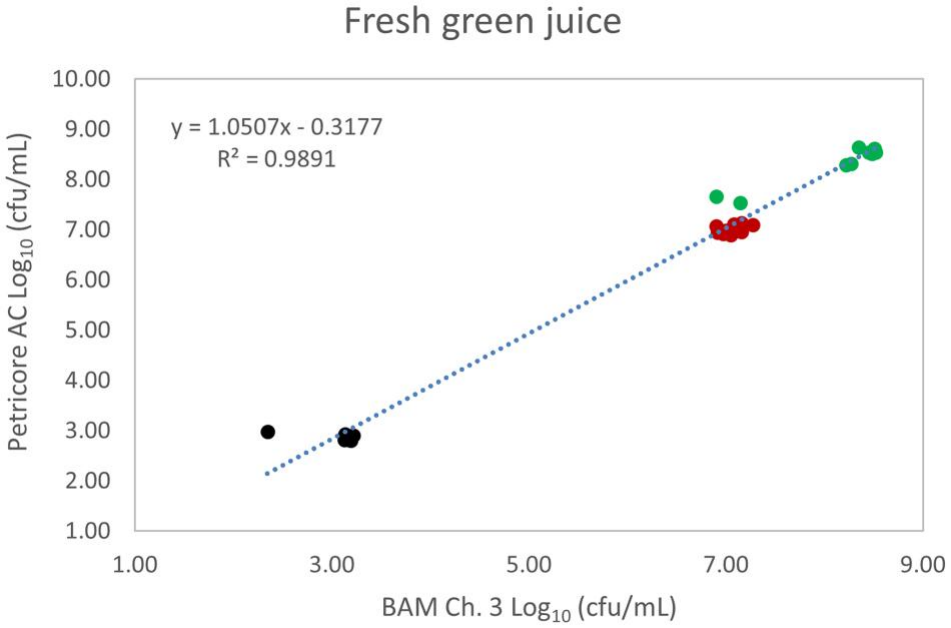
Petricore AC vs. BAM Ch. 3, fresh green juice.

**Figure 16.**
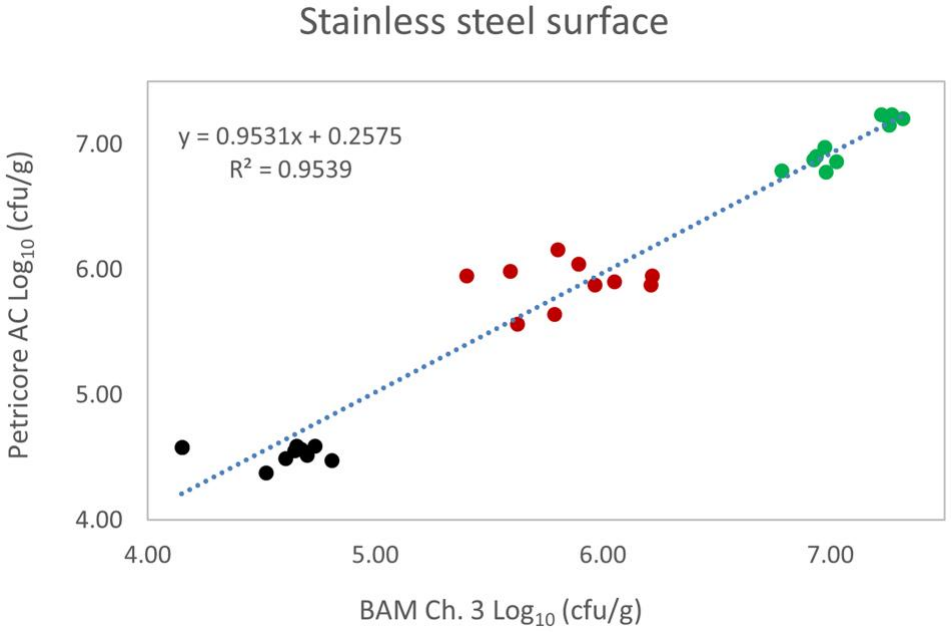
Petricore AC vs. BAM Ch. 3, stainless steel surface.

**Figure 17.**
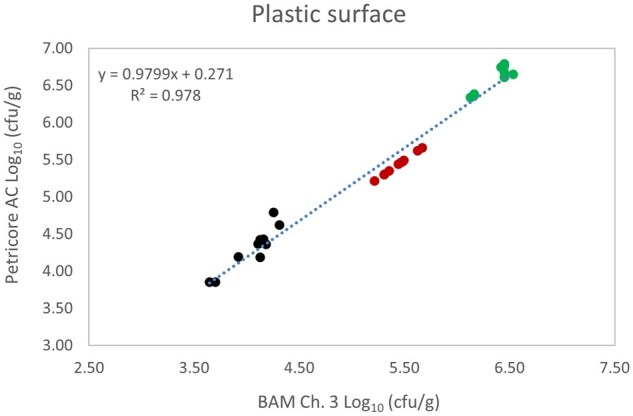
Petricore AC vs. BAM Ch. 3, plastic surface.

**Figure 18.**
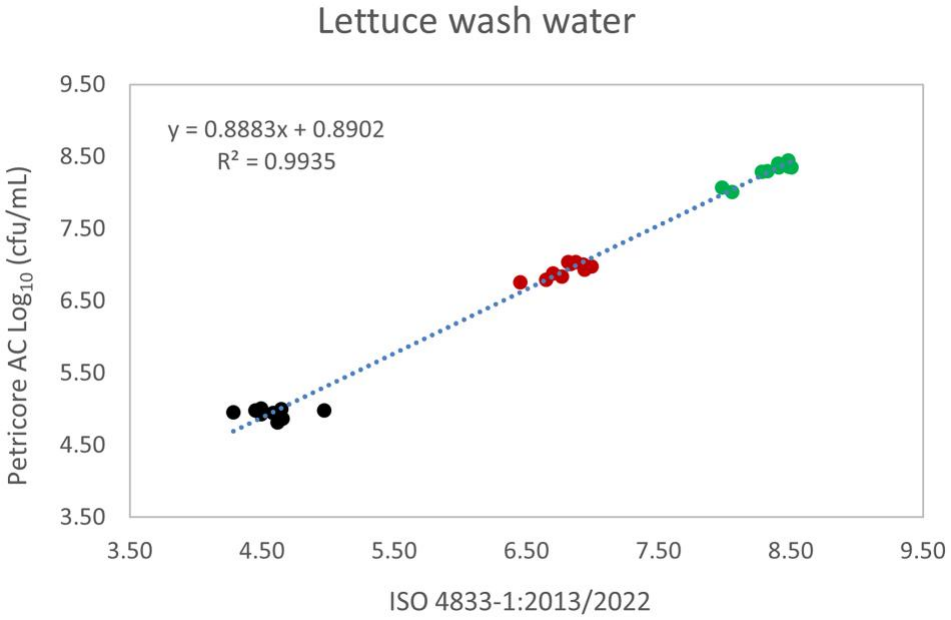
Petricore AC vs. ISO 4833-1:2013/2022, lettuce wash water.

**Figure 19.**
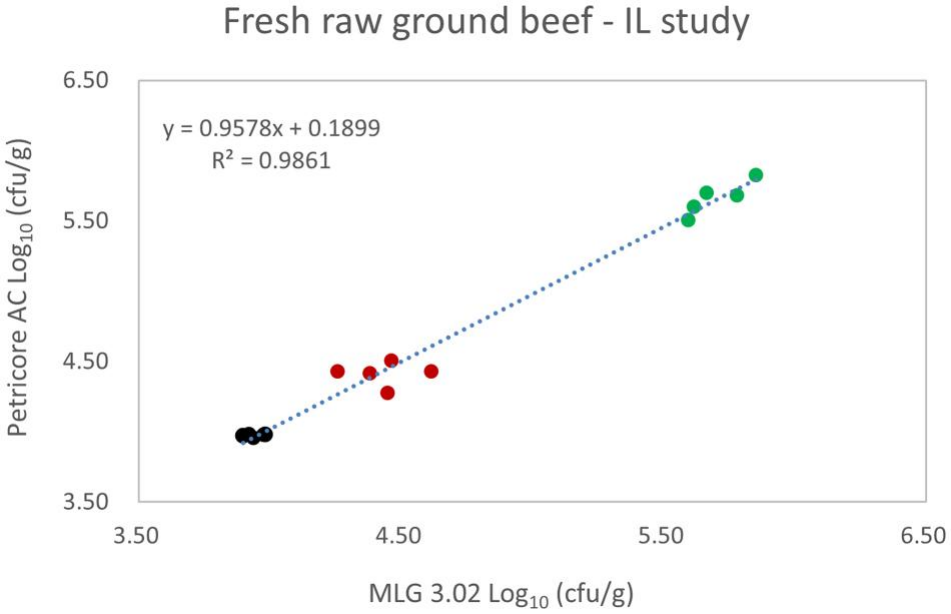
Petricore AC vs. MLG 3.02, fresh raw ground beef—independent laboratory study.

**Figure 20.**
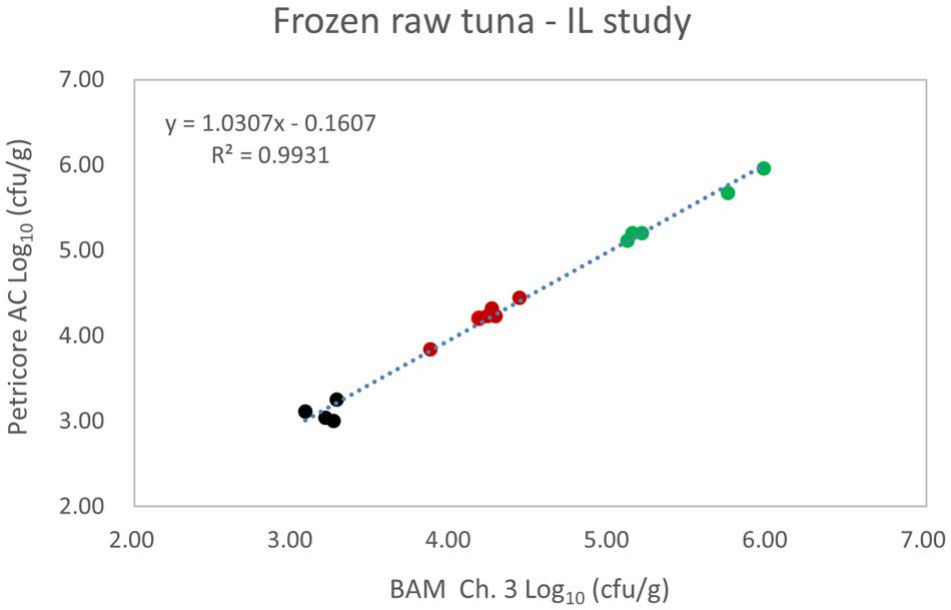
Petricore AC vs. BAM Ch. 3, frozen raw tuna—independent laboratory study.

**Figure 21.**
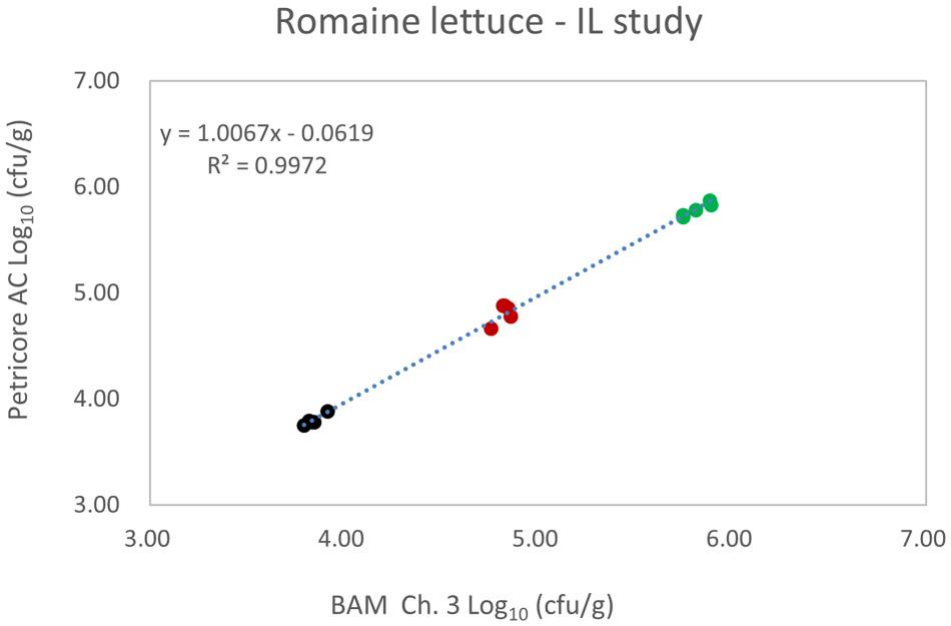
Petricore AC vs. BAM Ch. 3, Romaine lettuce—independent laboratory study.

**Figure 22.**
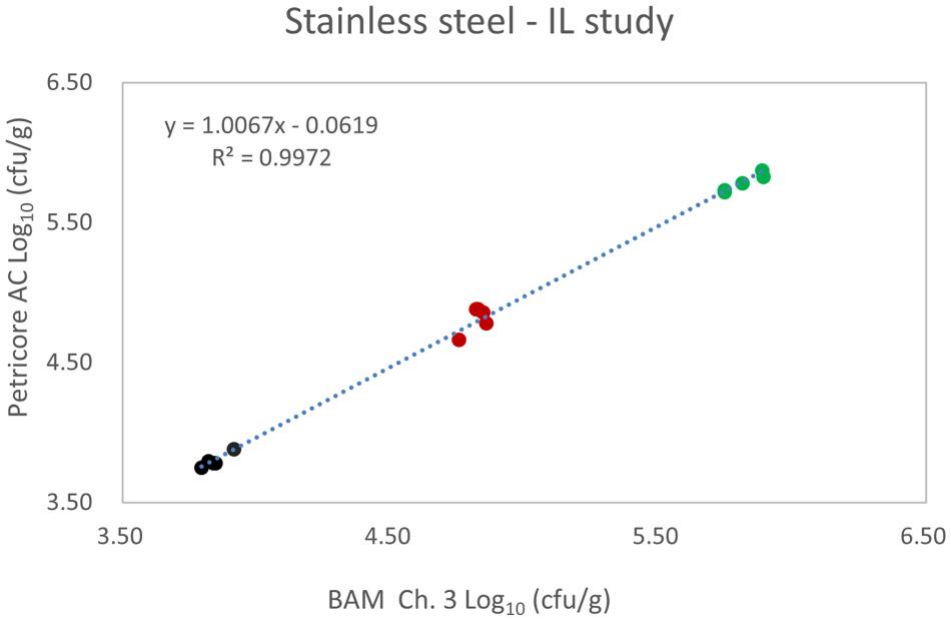
Petricore AC vs. BAM Ch. 3, stainless steel—independent laboratory study.

- *Matrix study.*—Results of the matrix studies are presented in Table 2. Scatter plots of the method comparisons are shown in Figures 1–22. For the food matrix test, the mean log_10_ differences between candidate method and reference methods ranged from −0.23 to 0.35 log_10_ within the acceptable range of −0.50 to 0.50 log_10._ The range of standard deviation values of the candidate method (0.01–0.88 log_10_) and the reference method (0.02–0.91 log_10_) were similar in 15 food matrixes. The lowest correlation factor (*R*^2^) was 0.9706 (frozen blueberries), which confirms that both methods are highly correlated.
  For the environmental matrixes, the mean log_10_ difference ranged from −0.17 to 0.26 log_10_ within the acceptable range of −0.50 to 0.50 log_10_. The range of standard deviation values of the candidate method (0.05–0.43 log_10_) and the reference method (0.04–0.43 log_10_) were similar in three environmental samples. The lowest correlation factor (*R*^2^) among three matrixes was 0.9539 (stainless steel surface), indicating strong correlation between the two methods. For all matrixes at both the method developer and independent laboratories, the 90% CIs of the bias (DOMs) between the Petricore AC and appropriate reference method were within ×0.5 to 0.5 log_10_ for all contamination levels, indicating equivalence between the methods (9). The Grubbs test was performed separately for the reference and the candidate methods to identify the highest variance. Among the 36 Grubbs test results, only 2 showed statistically significant outliers. The reference method in cooked sausage at the low level (log CFU/g 6.281) and frozen avocado at the high level (log CFU/g 5.127) were identified as outliers (*P *< 0.05). However, the data were not related to any known error or measurement disruption, these data points were retained for statistical analysis.
- *Product consistency/stability study.*—Results for the product consistency and stability study are shown in Table 3. Each of the three Petricore AC production lots (near expiration, middle of shelf life, and newly manufactured) were compared to the other using the artificially contaminated cooked sausage low and high levels, plus the non-inoculated level. The bias between lots was <0.05 log_10_ for all comparisons, and the 90% CIs were within −0.5 to 0.5 log_10_ in all cases, indicating that the production of the Petricore AC is consistent from lot-to-lot. The shelf life was established at 12 months.
- *Robustness study.*—Results of the robustness evaluation are presented in Table 4. The small variations in the test parameters (sample volume applied, incubation temperature, and incubation time) did not have a negative effect on the results. The bias between test combinations compared to the nominal test condition was <0.05 log_10_ for all comparisons, and the 90% CIs were within −0.5 to 0.5 log_10_ in all cases. All test combinations proved to be equivalent to the nominal test condition. Petricore AC plates are suitable for enumerating broad range of foods and select environmental samples; however, considering the physiological characteristics of microorganisms, a fixed incubation temperature and time (48 h) should be applied.

**Table 2.** Petricore AC: Matrix study results

Matrix/inoculating strain (if applicable)	Cont. level^a^	Petricore AC results		Reference method^d^ results		DOM^e^	SE^f^	90% CI^g^	95% CI^h^
		Mean^b^, log_10_ CFU/g	s_r_^c^	Mean, log_10_ CFU/g	s_r_				
Fresh raw ground pork	Low	3.76	0.14	3.92	0.07	−0.161	0.03	−0.213, −0.109	−0.225, −0.097
	Med	7.77	0.15	7.69	0.18	0.08	0.03	0.026, 0.125	0.014, 0.136
	High	8.37	0.08	8.38	0.06	−0.013	0.02	−0.043, 0.016	−0.049, 0.023
Fresh raw ground beef	Low	5.10	0.06	5.26	0.06	−0.16	0.01	−0.180, −0.140	−0.185, −0.136
	Med	7.61	0.14	7.60	0.12	0.01	0.07	−0.119, 0.137	−0.149, 0.167
	High	9.06	0.07	9.13	0.13	−0.07	0.03	−0.129, −0.001	−0.145, 0.014
Fresh raw ground beef^I^	Low	3.97	0.01	3.96	0.02	0.01	0.01	−0.014, 0.040	−0.022, 0.048
	Med	4.41	0.08	4.43	0.13	−0.02	0.07	−0.165, 0.127	−0.209, 0.171
	High	5.66	0.12	5.70	0.11	−0.04	0.03	−0.089, 0.016	−0.105, 0.032
Raw bacon	Low	6.02	0.37	5.98	0.36	0.01	0.03	−0.162, 0.186	−0.339, 0.363
	Med	7.37	0.25	7.34	0.29	0.03	−0.03	−0.019, 0.081	−0.031, 0.092
	High	8.56	0.25	8.50	0.24	0.06	0.03	0.003, 0.123	−0.011, 0.137
Raw shrimp	Low	4.24	0.14	4.47	0.25	−0.23	0.11	−0.421, −0.037	−0.466, 0.008
	Med	8.07	0.17	8.06	0.17	0.01	0.02	−0.034, 0.043	−0.044, 0.052
	High	8.86	0.07	8.79	0.20	0.07	0.07	−0.067, 0.203	−0.099, 0.234
Raw salmon	Low	7.62	0.11	7.45	0.11	0.17	0.04	0.100, 0.255	0.082, 0.273
	Med	8.62	0.16	8.46	0.24	0.16	0.05	0.075, 0.244	0.055, 0.264
	High	9.12	0.06	9.05	0.08	0.07	0.02	0.038, 0.101	0.031, 0.109
Frozen raw tuna	Low	2.71	0.37	2.80	0.31	−0.09	0.06	−0.196, 0.005	−0.220, 0.029
	Med	7.36	0.88	7.34	0.91	0.02	0.04	−0.053, 0.099	−0.072, 0.118
	High	8.02	0.53	7.96	0.68	0.07	0.07	−0.056, 0.187	−0.084, 0.215
Frozen raw tuna^I^	Low	3.20	0.23	3.34	0.31	−0.14	0.07	−0.280, −0.004	−0.321, 0.038
	Med	4.29	0.10	4.28	0.10	0.01	0.02	−0.028, 0.053	−0.040, 0.065
	High	5.43	0.37	5.44	0.40	−0.01	0.02	−0.052, 0.039	−0.066, 0.053
Frozen sliced mushrooms	Low	4.80	0.15	4.91	0.18	−0.11	0.04	−0.187, −0.039	−0.204, −0.022
	Med	8.79	0.15	8.79	0.14	0.00	0.03	−0.051, 0.029	−0.063, 0.072
	High	9.50	0.12	9.52	0.11	−0.02	0.03	−0.067, 0.029	−0.079, 0.041
Frozen avocado *Staphylococcus aureus* (ATCC^j^ 25923)	Non	0.000	NA^k^	0.000	NA	NA	NA	NA	NA
	Low	5.18	0.07	5.23	0.05	−0.05	0.07	−0.095, −0.012	−0.105, −0.002
	Med	6.17	0.04	6.17	0.04	−0.00	0.02	−0.032, 0.026	−0.039, 0.033
	High	7.06	0.05	7.17	0.10	−0.11	0.00	−0.185, −0.025	−0.204, −0.007
Frozen blueberries *Escherichia coli* (ATCC 25922)	Non	0.00	NA	0.00	NA	NA	NA	NA	NA
	Low	3.61	0.16	3.29	0.21	0.32	0.07	0.187, 0.451	0.156, 0.482
	Med	4.90	0.19	4.69	0.19	0.21	0.02	0.165, 0.248	0.156, 0.258
	High	5.65	0.11	5.62	0.14	0.03	0.05	−0.060, 0.117	−0.081, 0.138
Bacon-lettuce-tomato sandwich	Low	5.05	0.50	4.89	0.49	0.15	0.03	0.085, 0.205	0.071, 0.220
	Med	7.07	0.29	6.92	0.35	0.15	0.04	0.051, 0.099	0.046, 0.250
	High	8.48	0.58	8.33	0.35	0.16	0.06	0.050, 0.268	0.024, 0.294
Frozen pizza	Low	4.25	0.06	4.13	0.05	0.12	0.02	0.082, 0.165	0.072,0.175
	Med	7.12	0.09	6.77	0.07	0.35	0.03	0.287, 0.408	0.273, 0.422
	High	8.20	0.60	8.02	0.89	0.18	0.11	−0.023, 0.386	−0.071, 0.434
Cooked sausage *Salmonella* Typhimurium (ATCC 14028)	Non	0.00	NA	0.00	NA	NA	NA	NA	NA
	Low	4.07	0.05	4.14	0.03	0.06	0.01	0.045, 0.086	0.041, 0.086
	Med	5.15	0.03	5.07	0.04	0.08	0.01	0.046, 0.105	0.046, 0.105
	High	6.16	0.05	6.08	0.09	0.07	0.02	0.033, 0.115	0.023, 0.125
Romaine lettuce	Low	6.28	0.32	6.18	0.29	0.11	0.03	0.054,0.162	0.042, 0.175
	Med	7.84	0.16	7.81	0.18	0.04	0.02	−0.007, 0.080	−0.017, 0.090
	High	8.05	0.43	7.96	0.43	0.09	0.02	0.053,0.128	0.044, 0.137
Romaine lettuce^I^	Low	2.30	0.15	2.27	0.18	0.03	0.06	−0.101, 0.166	−0.142, 0.206
	Med	3.45	0.16	3.41	0.12	0.04	0.03	−0.027, 0.110	−0.047, 0.131
	High	5.18	0.07	5.10	0.08	0.08	0.04	−0.003, 0.168	−0.029, 0.194
Cabbage	Low	5.20	0.58	5.08	0.61	0.13	0.03	0.071, 0.186	0.057, 0.199
	Med	7.40	0.32	7.89	0.40	0.06	0.03	−0.014, 0.105	−0.028, 0.119
	High	8.15	0.32	8.16	0.04	−0.02	0.03	−0.065, 0.036	−0.076, 0.047
Fresh green juice	Low	2.88	0.06	3.08	0.26	−0.19	0.09	−0.361, −0.015	−0.041, 0.086
	Med	7.01	0.09	7.04	0.12	−0.03	0.04	−0.097, 0.034	0.046, 0.105
	High	8.33	0.40	8.13	0.60	0.19	0.07	0.057, 0.328	0.023, 0.125
Stainless steel surface *Listeria monocytogenes* (ATCC 7644) 4” × 4”	Non	0.00	NA	0.00	NA	NA	NA	NA	NA
	Low	4.54	0.07	4.64	0.10	−0.10	0.04	−0.168, −0.040	−0.184, −0.025
	Med	5.90	0.18	5.85	0.27	0.05	0.10	−0.131, 0.220	−0.172, 0.261
	High	7.01	0.19	7.07	0.18	−0.06	0.02	−0.108, −0.021	−0.118, 0.011
Stainless steel surface^I^ *Listeria monocytogenes* (ATCC 7644) 4” × 4”	Non	0.00	NA	0.00	NA	NA	NA	NA	NA
	Low	3.81	0.05	3.84	0.05	−0.03	0.01	−0.058, −0.008	−0.066, 0.000
	Med	4.81	0.09	4.82	0.04	−0.01	0.03	−0.079, 0.061	−0.100, 0.082
	High	5.79	0.06	5.82	0.07	−0.03	0.01	−0.050, −0.005	−0.057, 0.001
Plastic surface *Salmonella* Typhimurium (ATCC 14028) 4” x 4”	Non	0.00	NA	0.00	NA	NA	NA	NA	NA
	Low	4.31	0.30	4.05	0.23	0.26	0.04	0.187, 0.332	0.170, 0.349
	Med	5.27	0.15	5.44	0.14	−0.17	0.03	−0.226, −0.110	−0.239, −0.097
	High	6.57	0.19	6.33	0.16	0.24	0.02	0.194, 0.278	0.184, 0.288
Lettuce wash water	Low	6.28	0.32	6.18	0.29	0.11	0.03	0.054, 0.162	0.042, 0.175
	Med	7.84	0.16	7.81	0.18	0.04	0.02	−0.000, 0.080	−0.017, 0.090
	High	8.05	0.43	7.96	0.43	0.09	0.02	0.053, 0.128	0.044, 0.137

a All matrixes are artificially contaminated when a non-inoculated (Non) level is reported.

b Mean of five replicate portions, after logarithmic transformation: Log_10_[CFU/g + (0.1)f], where f is the reported CFU/unit corresponding to the smallest reportable result and unit is the reported unit of measure.

c s_r_ = Repeatability standard deviation.

d Reference methods are MLG 3.02 for fresh raw ground pork, fresh raw ground beef, raw bacon, and sausage; BAM Ch. 3 for raw shrimp, raw salmon, frozen raw tuna, frozen sliced mushrooms, frozen avocado, frozen blueberries, bacon-lettuce-tomato sandwich, frozen pizza, Romaine lettuce, cabbage, fresh green juice, stainless steel surface, and plastic surface; ISO 4833–1:2013/2022 for lettuce wash water.

e DOM = Difference of means between the Petricore AC method and the reference method.

f SE = Standard error on the difference of means between the Petricore AC and the reference method.

g 90% Confidence interval of the difference of means between the Petricore AC and the reference method. If the 90% CI falls within −0.5, 0.5, the results are equivalent.

h 95% Confidence interval of the difference of means between the Petricore AC and the reference method. If the 95% CI contains 0, the results are not statistically different.

I Matrix tested by the independent laboratory.

j American Type Culture Collection, Manassas, VA, USA.

k NA = Not applicable.

**Table 3.** Petricore AC**:** Product consistency and stability results

Matrix	Target level, CFU/g	*n* ^a^	Mean^b^, log_10_ CFU/g	s_r_^c^	Mean, log_10_ CFU/g	s_r_	DOM^d^	90% CI^e^	95% CI^f^
			**Lot A^g^ (near expiration date)**		**Lot B^h^(middle of the expiration date)**			**LCL^j^ UCL** ^**k**^	**LCL UCL**
Cooked sausage(*Salmonella* Typhimurium,ATCC 14028)	10^4^	5	6.04	0.05	6.01	0.06	−0.03	−0.07 0.01	−0.08 0.02
	10^2^	5	3.96	0.05	3.98	0.06	0.03	−0.01 0.06	−0.02 0.07
	0	5	<1.0	<1.0	<1.0	<1.0	<1.0	<1.0	<1.0
			**Lot A^g^(near expiration date)**		**Lot C^i^(recently manufactured)**			**LCL UCL**	**LCL UCL**
Cooked sausage (*Salmonella* Typhimurium, ATCC 14028)	10^4^	5	6.04	0.05	6.03	0.03	−0.01	−0.05 0.03	−0.05 0.04
	10^2^	5	3.96	0.05	3.98	0.05	0.03	−0.01 0.06	−0.02 0.07
	0	5	<1.0	<1.0	<1.0	<1.0	<1.0	<1.0	<1.0
			**Lot B(middle of the expiration date)**		**Lot C(Recently Manufactured)**			**LCL UCL**	**LCL UCL**
Cooked sausage (*Salmonella* Typhimurium, ATCC 14028)	10^4^	5	6.01	0.06	6.03	0.03	0.02	−0.01 0.05	−0.01 0.06
	10^2^	5	3.98	0.06	3.98	0.05	0.00	−0.04 0.04	−0.05 0.05
	0	5	<1.0	<1.0	<1.0	<1.0	<1.0	<1.0	<1.0

a *n* = Number of test portions.

b Mean of five replicate test portions (taken from the matrix study), after logarithmic transformation: Log_10_[CFU/g + (0.1)f], where f is the reported CFU/unit corresponding to the smallest reportable result and unit is the reported unit of measure.

c s_r_ = Repeatability standard deviation.

d DOM = Difference of means between the different Petricore AC lots tested.

e 90% Confidence interval of the difference of means between the Petricore AC and the reference method. If the 90% CI falls within −0.5, 0.5, the results are equivalent.

f 95% Confidence interval of the difference of means between the Petricore AC and the reference method. If the 95% CI contains 0, the results are not statistically different.

g Lot A, 12 months (near expiration date).

h Lot B, 6 months (middle of the expiration date).

i Lot C, 2 weeks (recently manufactured).

j LCL = Lower Confidence Limit.

k UCL = Upper Confidence Limit.

**Table 4.** Petricore AC: Robustness study results

Test combination	Test conditions				Test combination results		Nominal condition^d^ results				
	Sample volume, mL	Incubation time, h	Incubation temperature, °C	*n* ^a^	Mean^b^, log_10_ CFU/g	s_r_^c^	Mean, log_10_ CFU/g	s_r_	DOM^e^	90% CI^f^	95% CI^g^
Matrix study test portions (cooked sausage + *Salmonella Typhimurium* ATCC 14028)*—*low level											
1	0.95	44	33	5	4.057	0.040	4.071	0.048	−0.015	−0.056 0.027	−0.066 0.036
2	0.95	44	37	5	4.039	0.039	4.071	0.048	−0.032	−0.071 0.007	−0.080 0.016
3	0.95	52	33	5	4.044	0.047	4.071	0.048	−0.028	−0.069 0.014	−0.079 0.024
4	0.95	52	37	5	4.028	0.045	4.071	0.048	−0.043	−0.080 -0.006	−0.089 0.002
5	1.05	44	33	5	4.056	0.067	4.071	0.048	−0.016	−0.062 0.031	−0.073 0.042
6	1.05	44	37	5	4.091	0.053	4.071	0.048	0.024	−0.027 0.075	−0.039 0.088
7	1.05	52	33	5	4.082	0.060	4.071	0.048	0.010	−0.045 0.065	−0.058 0.078
8	1.05	52	37	5	4.040	0.053	4.071	0.048	−0.031	−0.074 0.012	−0.085 0.022
Matrix study test portions (cooked sausage + *Salmonella Typhimurium* ATCC 14028)*—*high level											
1	0.95	44	33	5	6.057	0.062	6.072	0.049	−0.014	−0.065 0.037	−0.077 0.049
2	0.95	44	37	5	6.057	0.062	6.072	0.049	−0.014	−0.062 0.034	−0.073 0.045
3	0.95	52	33	5	6.086	0.051	6.072	0.049	0.015	−0.032 0.062	−0.043 0.073
4	0.95	52	37	5	6.038	0.051	6.072	0.049	−0.034	−0.066 -0.002	−0.073 0.005
5	1.05	44	33	5	6.080	0.035	6.072	0.049	0.009	−0.021 0.038	−0.028 0.045
6	1.05	44	37	5	6.090	0.051	6.072	0.049	0.018	−0.019 0.056	−0.028 0.065
7	1.05	52	33	5	6.074	0.039	6.072	0.049	0.003	−0.026 0.032	−0.033 0.039
8	1.05	52	37	5	6.077	0.035	6.072	0.049	0.005	−0.032 0.043	−0.041 0.052
Matrix study test portions (cooked sausage + diluent)*—*blank											
1	0.95	44	33	5	<1.0	<1.0	<1.0	<1.0	<1.0	<1.0	<1.0
2	0.95	44	37	5	<1.0	<1.0	<1.0	<1.0	<1.0	<1.0	<1.0
3	0.95	52	33	5	<1.0	<1.0	<1.0	<1.0	<1.0	<1.0	<1.0
4	0.95	52	37	5	<1.0	<1.0	<1.0	<1.0	<1.0	<1.0	<1.0
5	1.05	44	33	5	<1.0	<1.0	<1.0	<1.0	<1.0	<1.0	<1.0
6	1.05	44	37	5	<1.0	<1.0	<1.0	<1.0	<1.0	<1.0	<1.0
7	1.05	52	33	5	<1.0	<1.0	<1.0	<1.0	<1.0	<1.0	<1.0
8	1.05	52	37	5	<1.0	<1.0	<1.0	<1.0	<1.0	<1.0	<1.0

a *n* = Number of test portions.

b Mean of five replicate test portions (taken from the matrix study), after logarithmic transformation: Log_10_[CFU/g + (0.1)f], where f is the reported CFU/unit corresponding to the smallest reportable result and unit is the reported unit of measure.

c s_r_ = Repeatability standard deviation.

d Nominal test condition = 1 mL sample volume, 48 h incubation time, and 35°C incubation temperature.

e DOM = Difference of means between the test combination parameters and the nominal test condition.

e 90% Confidence interval of the difference of means between the Petricore AC and the reference method. If the 90% CI falls within −0.5, 0.5, the results are equivalent.

f 95% Confidence interval of the difference of means between the Petricore AC and the reference method. If the 95% CI contains 0, the results are not statistically different.

## Conclusions

The study data have been evaluated in the AOAC Research Institute PTM Program and support certification of Petricore AC (AOAC PTM 032502) for enumeration of mesophilic aerobic bacteria within the scope indicated in Table 5. The performance of the Petricore AC method was successfully validated for the enumeration of mesophilic aerobic bacteria in all matrixes evaluated. No statistically significant differences were observed between the candidate and reference methods for all matrixes analyzed. The data from these studies, within their statistical uncertainty, support the product claims of the Petricore AC method for all matrixes evaluated. The Petricore AC method allows for fast and reliable enumeration of mesophilic aerobic bacteria. The plates are easy to interpret and also benefit from taking up less space in incubators. In addition, the method uses less media than standard pour plate techniques.

**Table 5.** Performance claims

Matrix	Test Portion			Incubation of plates		Reference method^b^	Claim^c^
		Diluent^a^	Volume (mL)	Temperature (°C)	Time (hours)		
Fresh raw ground pork	50 g	BPBD	450	35 ± 1	48 ± 2	MLG 3.02	Eq
Fresh raw ground beef	50 g	BPBD	450	35 ± 1	48 ± 2	MLG 3.02	Eq
Raw bacon	50 g	BPBD	450	35 ± 1	48 ± 2	MLG 3.02	Eq
Raw shrimp	50 g	BPBD	450	35 ± 1	48 ± 2	BAM Ch. 3	Eq
Raw salmon	50 g	BPBD	450	35 ± 1	48 ± 2	BAM Ch. 3	Eq
Frozen raw tuna	50 g	BPBD	450	35 ± 1	48 ± 2	BAM Ch. 3	Eq
Frozen sliced mushrooms	50 g	BPBD	450	35 ± 1	48 ± 2	BAM Ch. 3	Eq
Frozen avocado	50 g	BPBD	450	35 ± 1	48 ± 2	BAM Ch. 3	Eq
Frozen blueberries	50 g	BPBD	450	35 ± 1	48 ± 2	BAM Ch. 3	Eq
Bacon-lettuce-tomato sandwich	50g	BPBD	450	35 ± 1	48 ± 2	BAM Ch. 3	Eq
Frozen pizza	50 g	BPBD	450	35 ± 1	48 ± 2	BAM Ch. 3	Eq
Cooked sausage	50 g	BPBD	450	35 ± 1	48 ± 2	MLG 3.02	Eq
Romaine lettuce	50 g	BPBD	450	35 ± 1	48 ± 2	BAM Ch. 3	Eq
Cabbage	50 g	BPBD	450	35 ± 1	48 ± 2	BAM Ch. 3	Eq
Fresh green juice	50 mL	BPBD	450	35 ± 1	48 ± 2	BAM Ch. 3	Eq
Stainless steel surface	4” × 4” in^2^	BPBD	90	35 ± 1	48 ± 2	BAM Ch. 3	Eq
Plastic surface	4” × 4” in^2^	BPBD	90	35 ± 1	48 ± 2	BAM Ch. 3	Eq
Lettuce wash water	50 mL	BPBD	450	35 ± 1	48 ± 2	ISO 4833-1:2013/2022	Eq

a BPBD = Butterfield’s phosphate buffer diluent.

b BAM = U.S. Food and Drug Administration *Bacteriological Analytical Manual*; MLG = U.S. Department of Agriculture Food Safety and Inspection Service *Microbiology Laboratory Guidebook*; ISO = International Organization for Standardization.

c Eq = Equivalence of candidate and reference methods demonstrated by 90% confidence interval of difference of means contained entirely within −0.5 to 0.5 log_10_ CFU/g.
